# Analysis and correcting pronunciation disorders based on artificial intelligence approach

**DOI:** 10.3389/frai.2025.1388180

**Published:** 2025-06-05

**Authors:** Nataliia Melnykova, Bohdan Pavlyk, Oleh Basystiuk, Stepan Skopivskyi

**Affiliations:** Department of Artificial Intelligence, Lviv Polytechnic National University, Lviv, Ukraine

**Keywords:** pronunciation, unclear pronunciation, dysarthria, classification, CNN, LSTM, artificial intelligence tools

## Abstract

The main aim of this study is to employ artificial intelligence and machine learning methods to assess and correct pronunciation disorders in post-traumatic military patients, acknowledging the critical need for effective communication rehabilitation in individuals who have experienced trauma, such as head injuries or war-related incidents. Tasks include reviewing existing research, selecting appropriate machine learning methods, generating relevant training data, and implementing a software architecture tailored to analyze and correct pronunciation defects in this specific population. The analysis of machine learning methods led to the selection of two experimental models: a Convolutional Neural Network (CNN) utilizing mel-spectrograms for image-based sound representation and a Long Short-Term Memory (LSTM) network combined with mel-frequency cepstral coefficients, aiming to explore the effectiveness of sequential data processing in the context of pronunciation disorder classification in post-traumatic military patients. The results of the two models were compared based on the loss and accuracy functions of the training and validation data, error matrices, and such key metrics as precision, recall, and F1-score. Both models showed promising results in classifying dysarthria stages, but the CNN model performed slightly better in predicting all classes than the LSTM.

## 1 Introduction

Technological evolution is relatively rapid, and now, many processes and duties performed by humans can be replaced by computers; in many professions, artificial intelligence can easily replace humans, including in medicine. In these difficult times, our country has faced many challenges that must be addressed immediately. One of the most critical tasks is to save the lives of every Ukrainian, for which the military suffers injuries on the battlefield that can last a lifetime. One of these problems can be a speech impediment. In such cases, automatic voice analysis could help the military rehabilitate faster. It will also be helpful for post-traumatic patients who have problems with speech for some reason.

The relevance of this topic is due to the problematic situation in the country, as pronunciation disorders can occur in military personnel who have suffered trauma, such as head injuries or war. This also supports the practical value, as research in this area can help military personnel who have suffered trauma to improve their pronunciation and make their speech more understandable to others.

This research aims to find practical solutions for developing a software module to provide analysis, selection of artificial intelligence tools, and verification of the effectiveness of machine learning methods in classifying stages of disease for post-traumatic patients.

The object of study is the process of analysis and correction of pronunciation in post-traumatic patients, and the subject is the voice parameters of post-traumatic military patients for speech defects and the artificial intelligence method for analysis and correction of pronunciation in post-traumatic patients.

## 2 Current research analysis

Artificial intelligence in medicine is already commonplace today, and this trend will continue to grow. The relevance of this topic can also be confirmed by the situation in our country, where every second counts.

The importance of this issue is associated with the following key factors:

- Expansion of rehabilitation capabilities: Post-traumatic speech disorders are a significant problem that arises after various types of brain injuries (e.g., stroke or traumatic brain injury). Patients facing these problems often require long-term rehabilitation, and artificial intelligence can provide additional tools to support this process. AI-driven speech recognition systems can analyze patients’ speech patterns in real-time, identifying pronunciation errors and providing feedback (Abdulmajeed et al., 2022).
- Accessibility and convenience: Artificial intelligence technologies can allow patients to engage in rehabilitation at a convenient time and place, improving their ability to recover (Lu et al., 2020).
- Adaptability: Artificial intelligence can adapt to the individual needs of each patient, providing more personalized and effective interventions.
- Monitoring and tracking progress: By using artificial intelligence methods, medical professionals can better track patients’ progress in real time, allowing them to adjust treatment plans as needed (Rastogi, 2020).
- Resources: In a world with a constantly increasing number of patients and limited medical resources, artificial intelligence can be an effective means of maximizing the use of these resources.
- Improvement of treatment outcomes: Artificial intelligence can help find new approaches to speech exercises that can accelerate recovery (Duda, 2025).
- Classification of speech problems: Recognition and classification of different types of speech disorders are crucial elements in this field. Speech involves various aspects, including articulation, tempo, pitch, and rhythm. Artificial intelligence can assist in analyzing and classifying these complex speech characteristics. Such classification will enable medical professionals better to understand the specifics of the patient’s problem and tailor rehabilitation programs for maximum effectiveness (Deruty, 2025).

The PRISMA scheme was employed to systematically analyze the relevant literature, ensuring a comprehensive and transparent approach to source selection. This process provided a foundation for identifying key studies that address pathology detection through advanced analytical methods.

In article by Panek et al. (2015), issues related to pathology detection are addressed by creating a feature vector consisting of 28 different parameters obtained through voice signal analysis. Based on this, the accuracy and specificity of pathology detection using machine learning methods such as principal component analysis (PCA), kernel principal component analysis (kPCA), and auto-associative neural network are compared. The experimental results show that the PCA methodology effectively reduced the data, retaining 90% of the variance. However, PCA yielded slightly better results than PCA, albeit with a longer computation time due to the increased number of parameters.

In the subsequent article by Hossain and Muhammad (2016), a framework for big data in healthcare is proposed, utilizing voice pathology assessment (VPA) as an example. The VPA system employs two reliable functions, MPEG7 low-level audio and template derivative cepstral analysis, for processing voice or speech signals.

Combining Incremental Discriminant Analysis (IDP) functions and the Extreme Learning Machine (ELM) classifier yielded the most accurate (95%) and fastest (slightly over a second processing time) results. However, the limited training data and the narrow selection of voice samples are highlighted as drawbacks.

Verde et al.’s (2018) objective was to determine an algorithm that can differentiate between pathological and healthy voices with higher accuracy, which is necessary for implementing a practical and precise mobile healthcare system. Analyses were conducted on a large dataset of 1,370 voices selected from the Saarbrucken Voice database, with testing performed on both the entire dataset and three different subsets. Support Vector Machine (SVM) algorithm achieved the highest accuracy in detecting voice pathology (86%). However, the insufficient data processing speed (time from incoming data to decision received) is a drawback, which could be addressed using multiple machine learning methods.

Alhussein and Muhammad (2018) investigate a voice pathology detection system using deep learning on a mobile healthcare system. A mobile multimedia healthcare system was developed using smart mobile devices to record voices. In experiments with the SVD database, the system achieved 98.77% accuracy using the CaffeNet CNN model, followed by the SVM classifier. The lack of recognition speed indicator in the study is a drawback, as it is crucial for the system’s optimal performance.

Alhussein and Muhammad (2019) proposes using an intelligent healthcare system on a mobile platform utilizing deep learning. The smartphone records the client’s voice signal and sends it to a cloud server, which processes and classifies it as normal or pathological using a parallel convolutional neural network model. The decision on the signal is then transmitted to the doctor for a prescription. Experimental results on the SVD database showed that the proposed system achieved 95.5% accuracy, with over 95% accuracy in pathology classification. A 3-layer MLP fusion outperformed 2-layer MLP and ELM-based fusion. However, unlike the previous article, the absence of decision-finding speed is highlighted as another drawback.

In work by Al-Dhief et al. (2020), an extensive review of the latest methods and research frameworks of IoT and machine learning algorithms used in healthcare, particularly in voice pathology surveillance systems, is presented. This work outlines the latest strategies and research frameworks, discussing their applications in healthcare systems. It also identifies key challenges, such as data privacy concerns, system interoperability, and the computational limitations of IoT-enabled systems. The insights from this review are directly relevant to the current study, as they provide a foundational understanding of the broader context in which voice pathology detection frameworks operate. The authors also discuss the applications, challenges, and key issues of IoT and machine learning algorithms in healthcare.

Tuncer et al. (2020) aim to present a multi-class pathological voice classification using a novel multilevel texture feature extraction with an iterative feature selector. This simple and effective voice algorithm utilizes a multi-center and multi-threshold triple template (MCMTTP). A set of pathological voice data from SVD was used to create 8 cases, focusing on three disorders—cordectomy, frontal resection, and spastic dysphonia. This method achieved 100.0% classification accuracy and geometric mean (ideal classification) for the case of detecting frontal resection. The proposed MCMTTP and INCA-based methods demonstrated high efficiency. However, a limitation is identified as testing this solution only on three disorders, making it less universal and possibly compromising accuracy with increased data.

In article by Al-Dhief et al. (2021), a system for detecting and classifying voice pathology using the OSELM algorithm as a classifier and Mel-frequency cepstral coefficients (MFCC) as feature extraction is presented. Voice samples were taken from the Saarbrucken Voice database (SVD). This system comprises two parts of the database; the first part includes all voices in SVD with sentences and vowels /a/, /i/, and/u/ pronounced at high, low, and normal pitches. The second part uses voice samples of three common pathologies (cyst, polyp, and paralysis) based on the vowel /a/, produced with standard pitch. Experimental results demonstrate that the OSELM algorithm can distinguish healthy and pathological voices with a maximum accuracy of 91.17%.

Islam and Tarique (2022) introduces an algorithm for pathological voice detection based on a convolutional neural network (CNN) using a signal processing approach. The proposed algorithm extracts an acoustic characteristic called a chromatogram from voice samples and applies this feature to the CNN input for classification. Using chromatograms achieves higher accuracy than other unique characteristics (71% versus 85% accuracy). Different performance parameters, including precision, recall, and F1 score, also confirm the effectiveness of the proposed algorithm. A drawback could be the insufficient experimentation, specifically with chromatograms in combination with other machine learning methods, with only a convolutional neural network.

Nandi (n.d.) gathered recent research, voice pathology detection methods, machine learning, and deep learning methods (DL) used for data classification, presenting various applications, open challenges, and recommendations for future directions of IoT systems and artificial intelligence (AI) approaches in diagnosing voice pathology. Examples of machine learning methods such as Kay Pentax CSL Model, GMM, SVM, CNN, DPM, RNN, OSELM, ResNet50, Xception, and MobileNet are provided.

From the analysis of scientific sources and the relevance of the work, the topic of voice pathology detection is widespread and attracts considerable attention. The most effective methods for addressing this problem are convolutional neural networks (CNN) and Gaussian mixture models (GMM). However, there are several main drawbacks:

- Insufficient sampling of voice data (for example, the existence of various accents can complicate program operation).
- Slow training and recognition times.
- Limited availability of universal datasets.

The above-mentioned drawbacks lie in finding a qualitative and comprehensive dataset for accurate model training and improving system performance times. Most articles do not provide comparative results of training and subsequent recognition speed by neural networks, so this factor needs to be considered for further selection of artificial intelligence tools.

## 3 Materials and methods

### 3.1 Machine learning models

Two neural models are being used in this paper. The first one is a convolutional neural network. The model is initialized as a sequential model. A sequential model is a linear stack of layers easily created by passing a list of layer instances to the constructor. The first layer is the 2D convolution layer (Conv2D). This layer creates a convolution kernel with the layer’s input data to generate the output tensor. The first argument, “32,” is the number of output filters in the convolution. The kernel size indicates the height and width of the 2D convolution window; here, it equals (3,3). The “real” activation function is applied to the original data. The next layer is the MaxPooling2D layer with a pool size of (2,2). This layer applies the maximum pooling operation to the input data, reducing its dimensionality. The Dropout layer with a dropout rate of 0.25 randomly “switches off” 25% of the neurons during each update during training, which helps prevent overtraining.

**Figure 1:**
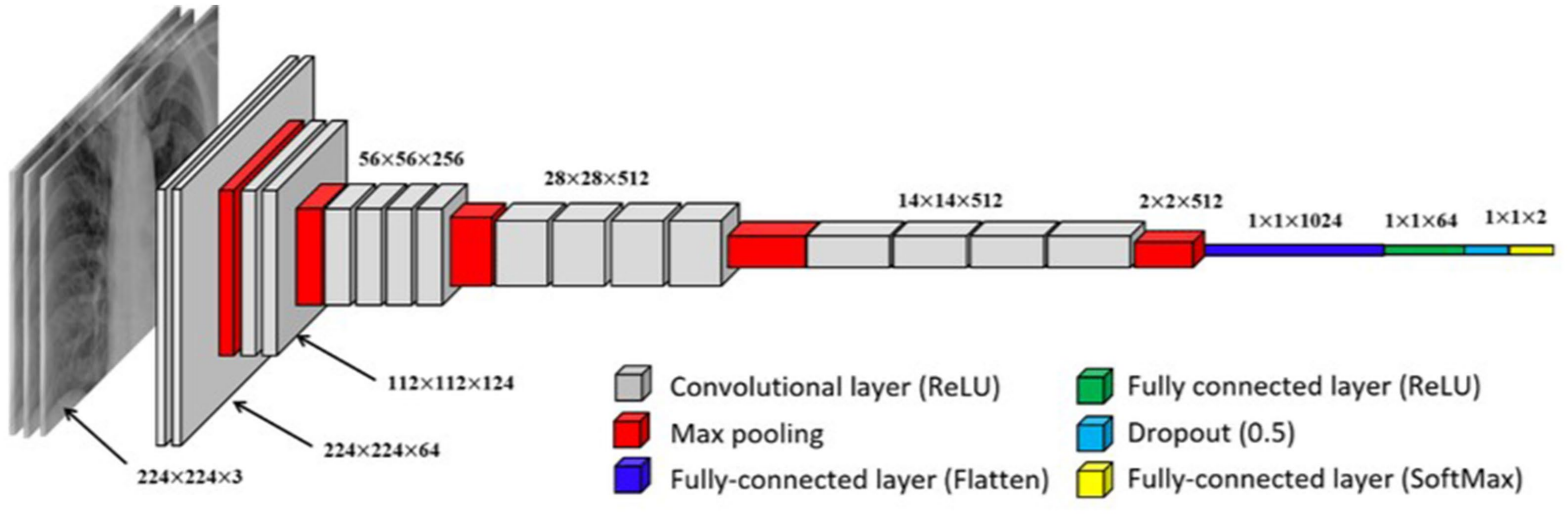
Visual representation of a convolutional neural network (CNN).

These layers (Conv2D, MaxPooling2D, Dropout) are repeated once more, but this time the Conv2D layer has 64 output filters. The Flatten layer converts the previous layer’s output into a one-dimensional array. This is necessary to enter the data into the fully connected layer (Dense). The Dense layer with 128 neurons performs a dot operation on the output data and layer weights and then adds the offset. This is a fully connected layer. Another Dropout layer with a rejection rate of 0.25 is added after the fully connected layer. The last layer, Dense with 4 neurons, uses the activation function “softmax,” which converts the output data into probabilities of 4 classes. The sum of these probabilities for all classes is 1 (see Figure 1).

**Figure 2:**
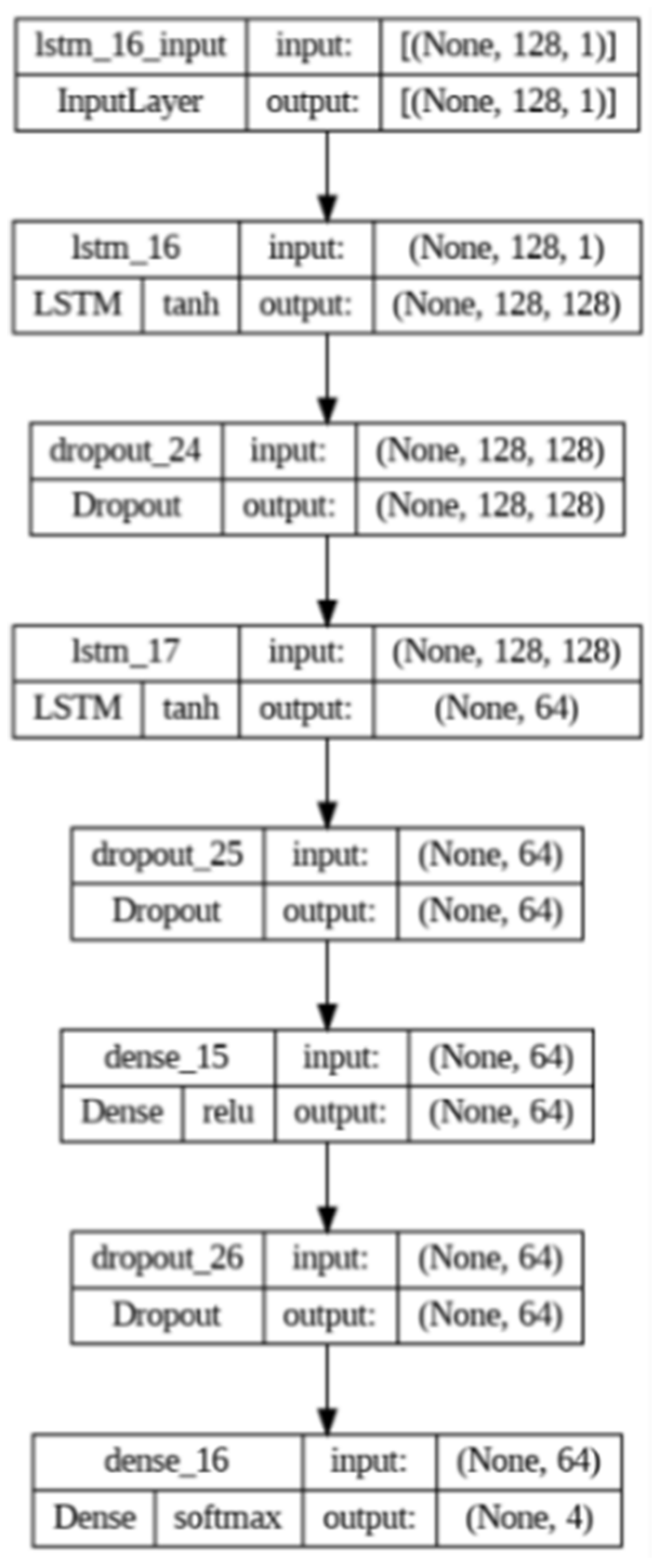
Detailed diagram of the LSTM network.

The Long Short-Term Memory (LSTM) model selected for the experiments is a recurrent neural network (RNN) designed to handle sequential data efficiently. LSTM is particularly well-suited for tasks involving time-dependent patterns, such as speech recognition, due to its ability to retain long-term dependencies in data. A schematic representation of the LSTM model is shown in Figure 2.

This model includes seven layers:

1. The first LSTM layer: This layer includes 128 LSTM units. LSTM (Long Short-Term Memory) is a type of recurrent neural network (RNN) that efficiently processes sequential data by retaining a “memory” of previous steps. In this model, the first LSTM layer returns the output for each time step, which is essential for the next LSTM layer.
2. First Dropout layer: This layer applies a random dropout technique that helps reduce overtraining by randomly disconnecting a specified fraction (here, 30%) of the input neurons at each training step.
3. The second LSTM layer: This layer contains 64 LSTM units. It receives sequential outputs from the previous LSTM layer. Only the last production in the time sequence is returned in this layer.
4. Second Dropout layer: Similar to the first, this layer turns off 30% of the neurons to prevent overlearning.
5. Dense layer: This layer contains 64 units and uses the ReLU (Rectified Linear Unit) activation function. The ReLU function is very popular in deep learning because it adds the necessary nonlinearity to the model without affecting the learning speed.
6. The third Dropout layer: Another random dropout layer with a 30% level.
7. Output dense layer: This layer has four units corresponding to the number of classes in the classification problem. The layer uses softmax’s activation function, which converts the output values into probable.

### 3.2 Audio feature representation

The Mel Frequency Cepstral Coefficients (MFCC) and Mel Spectrograms are characteristics derived from audio signals, and both are based on the Mel scale, a perceptual pitch scale closely related to human auditory perception. However, they represent an audio signal differently and are used for different purposes.

#### 3.2.1 Data representation

A Mel spectrogram is a visual representation of an audio signal. It shows how the power spectrum of an audio signal is distributed at different frequencies over time. Each point on the Mel spectrogram corresponds to the power at a specific time and frequency.

MFCC is a numerical representation of an audio signal. It provides a compact representation of the power spectrum of an audio signal, focusing on the aspects most relevant to human perception. MFCC is typically represented as a sequence of feature vectors (one per time slot).

#### 3.2.2 The process of feature extraction

Mel spectrogram: To compute the Mel spectrogram, the audio signal is segmented into short frames, the power spectrum for each frame is calculated, a group of Mel filters is applied to the power spectra, and then the logarithm of the filter group energies is taken.

MFCC: The process of calculating MFCC is similar to the process for Mel spectrograms, but there is one additional step. After logarithmizing the filter bank energies, a discrete cosine transform (DCT) is applied to decorelate the energies and reduce the dimensionality of the data.

#### 3.2.3 Usage cases

Mel spectrograms are often used as input to convolutional neural networks (CNNs) for audio classification tasks because they provide a two-dimensional representation of an audio signal that can be viewed like an image.

MFCC: used as features in a wide range of audio and speech processing tasks, including speech recognition, speaker identification, and music genre classification. They are often used as input to models that can process sequential data.

After analyzing everything, we decided to experiment with two machine-learning models:

– CNN using the mel-spectrogram, since in this case, there will be a representation of sound in the form of images, which the convolutional network can perfectly cope with the classification of
– LSTM in combination with fine-frequency cepstral coefficients to test the model’s performance with sequential data.

### 3.3 Dataset

In this study, the TORGO dataset, designed explicitly for dysarthria research, was used from Kaggle. The TORGO dataset collected data from seven individuals with different forms and severities of dysarthria. Each speaker was asked to complete several speaking tasks, which included:^1^

- Saying a set of regular phrases is commonly used in everyday speech and indicates how people with dysarthria say typical statements.
- Reading a phonetically balanced set of sentences: this task uses a standardized text often used in speech and language research. By asking speakers to read this passage, researchers can collect data on how people with dysarthria produce a wide range of phonemes in a controlled context.
- Single-word pronunciation: This task provides insight into how speakers pronounce individual words, which can be particularly useful for examining specific aspects of dysarthria that may be less apparent in continuous speech.

The disease classification in this dataset is shown in Table 1.

**Table 1:** Disease classes in the dataset.

Degree of the disease	Data
Healthy (or 1 degree of illness)	FC03, FC04, MC03, FC01, MC05, MC01, MC02, MC04
2nd degree	F03, F04, M03
3rd degree	F01, M05
4th degree	M01, M02, M04

**Figure 3:**
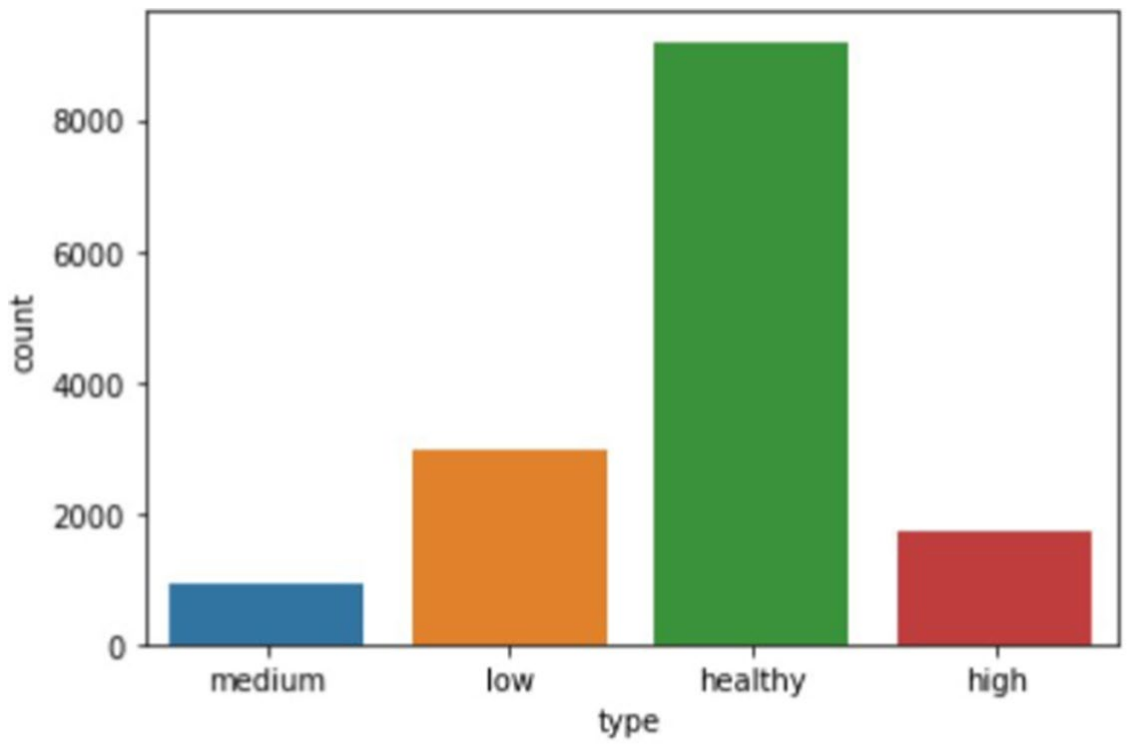
Bar chart showing the number of audio recordings of each stage of the disease.

The visualization of the disease classes is shown in Figure 3.

## 4 Results

A software module was developed and implemented in Python to obtain the research results, widely used for machine learning development. Python has a rich ecosystem of scientific libraries, including powerful libraries for machine learning and data analysis, such as TensorFlow, PyTorch, ScikitLearn, Pandas, NumPy, and Matplotlib.

The research on the selected dataset (see text footnote 1) was conducted using CNN with mel-spectrograms and LSTM with MFCC.

CNNs use images as input, but they have been trained and evaluated on large datasets with various classes for good results. Examples of such sets are ImageNet (a database containing about 14 million images), Open Images (9 million images), and others.

Sound signals contain valuable information that changes over time, requiring models to account for long-term dependencies. One way to solve this problem is to combine CNNs with recurrent neural networks (RNNs), such as long-short-term memory (LSTM) networks. By integrating CNN and LSTM layers, CNN-LSTM networks can take advantage of the strengths of both architectures: efficiently extracting spatial features from spectrograms while capturing temporal dynamics.

Long short-term memory (LSTM) networks are recurrent neural networks (RNNs) widely used in sequence modeling tasks due to their ability to capture long-term dependencies. LSTMs solve the vanishing gradient problem that traditional RNNs face, allowing them to learn and store information in long sequences efficiently. A key point in the LSTM architecture is the inclusion of memory cells and gate mechanisms that regulate the flow of information. These mechanisms consist of three main gates: input, forgetting, and output.

- The input gate determines the new information added to the memory cell. It takes the current input signal and the previous hidden state as input and passes them through a sigmoid activation function.
- The forgetting gate controls the amount of information removed from the memory cell.
- The output gate regulates the amount of information output from the memory cell. Like a forget-me-not gate, it takes the current input and the previous hidden state as inputs and passes them through a sigmoid activation function. The resulting activation output vector is multiplied element by element with the state of the memory cell and passed through the hyperbolic tangential activation function (tanh) to obtain the output of the LSTM block.

**Figure 4:**
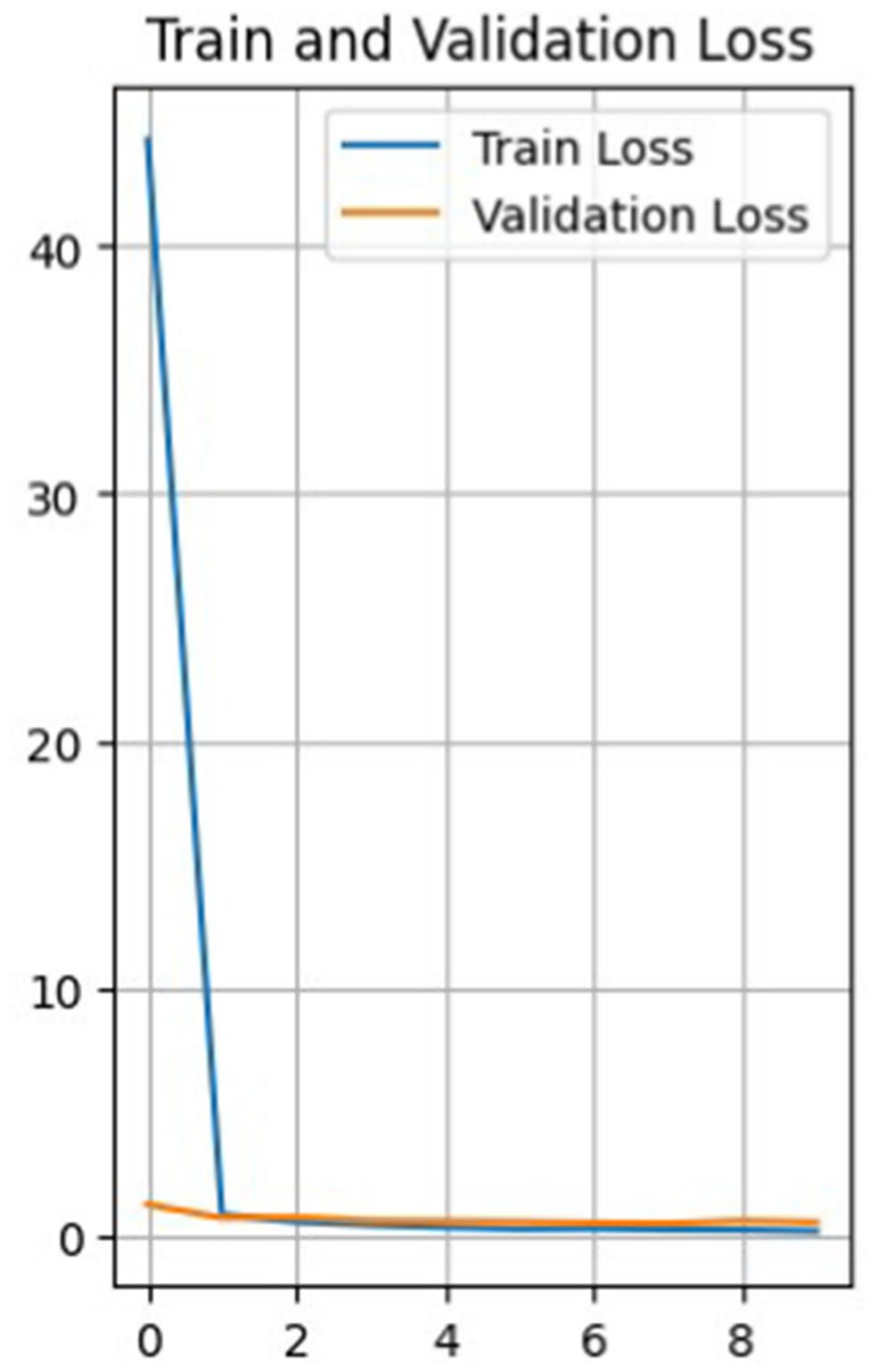
Graph of the loss function of validation and training data versus epoch.

So, Figure 4 shows a plot of the loss function of the training and test data versus the epoch before the model. The training loss function decreases with each epoch, starting from a value of about 44 in the first epoch and reaching about 0.16 in the last epoch, the 10th epoch. This indicates that the model learns and improves its predictions with each epoch.

The loss function for validation also decreases with each epoch, but not as uniformly. It first decreases from 1.24 to 0.70 from epoch 1 to 2, then increases to 0.72 in epoch 3, and then decreases again to 0.49 in epoch 7. It then increases again to 0.57 in the 9th epoch and decreases to 0.52 in the 10th.

Such fluctuations in the loss on the validation set may indicate that the model has some level of overfitting, as it shows a more significant error on the validation set than on the training set. It can also result from noise in the data or heterogeneity in the validation set.

Nevertheless, the model improves overall as training and validation losses are reduced. The accuracy of the training data constantly increases from epoch to epoch, with a final accuracy of 0.946, while the accuracy of the validation data does not increase as smoothly.

**Figure 5:**
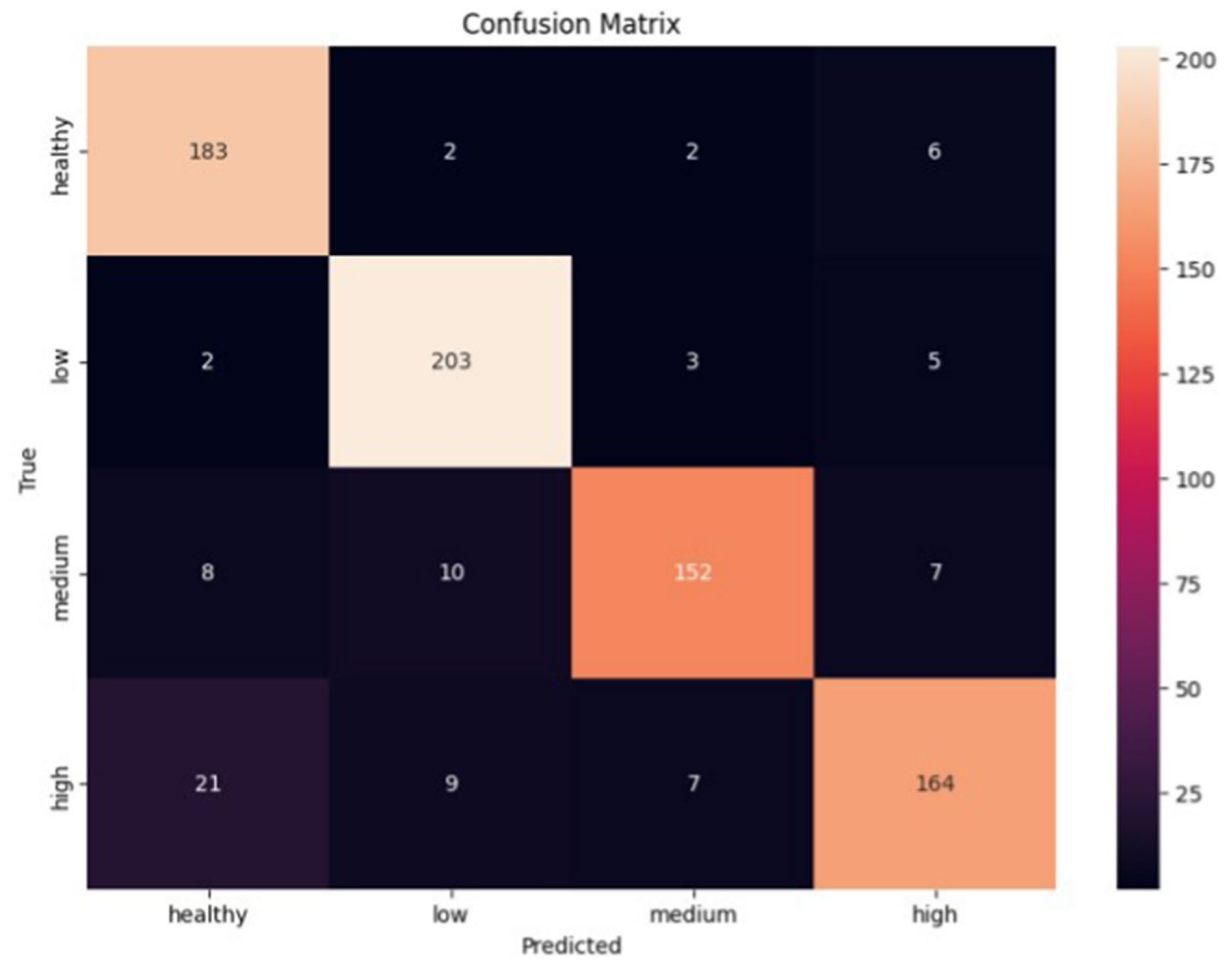
Error matrix of convolutional neural network.

Figure 5 shows the error matrix of the convolutional network. Based on it, the classifier made the most errors between the health and high classes, while the low class was the best predicted. Given the errors, the classifier showed promising results in classifying all classes.

All classes have high precision, recall, and F1-score metrics scores. However, there are some differences. The ‘Healthy’ and ‘Low’ classes have high values for both the precision and recall parameters, which indicates that the model can correctly identify and predict these classes. On the other hand, although the precision parameter is high for the ‘Medium’ and ‘High’ classes, the recall is slightly lower compared to the ‘Healthy’ and ‘Low’ classes. This may indicate that the model has more difficulty correctly predicting these classes.

**Figure 6:**
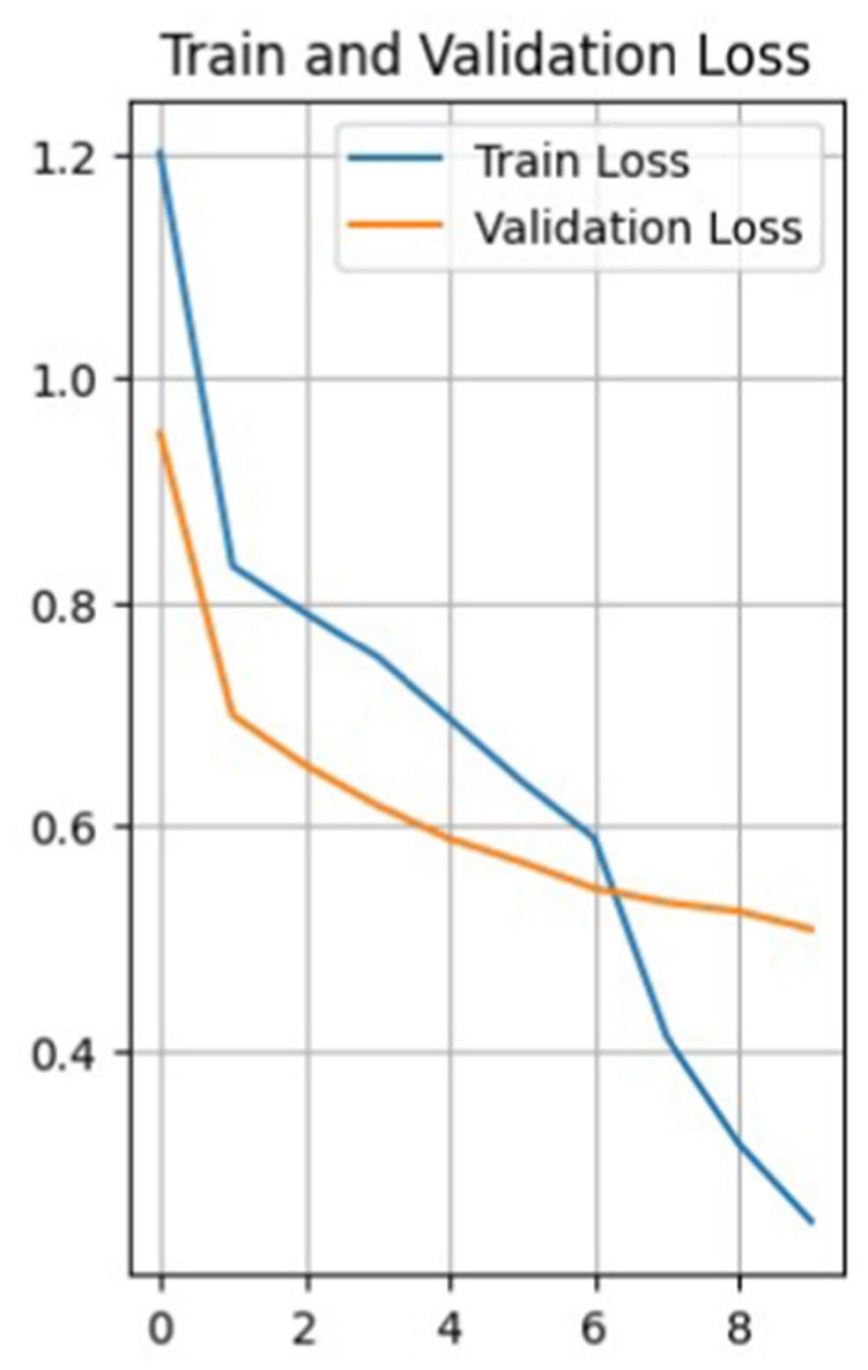
Validation and training data loss graph.

As for the LSTM model, the results of the experiments are presented below:

Figure 6 shows a graph of data loss. The model shows a decrease in the loss function with each epoch, both on the training and validation samples. This is a good indicator, showing that the model is learning and reducing prediction errors. The loss function on the training data decreases from 1.2001 in the first epoch to 0.2480 in the tenth epoch, and the loss function on the validation data gradually decreases, indicating that the model is also performing well on data it has not seen before.

**Figure 7:**
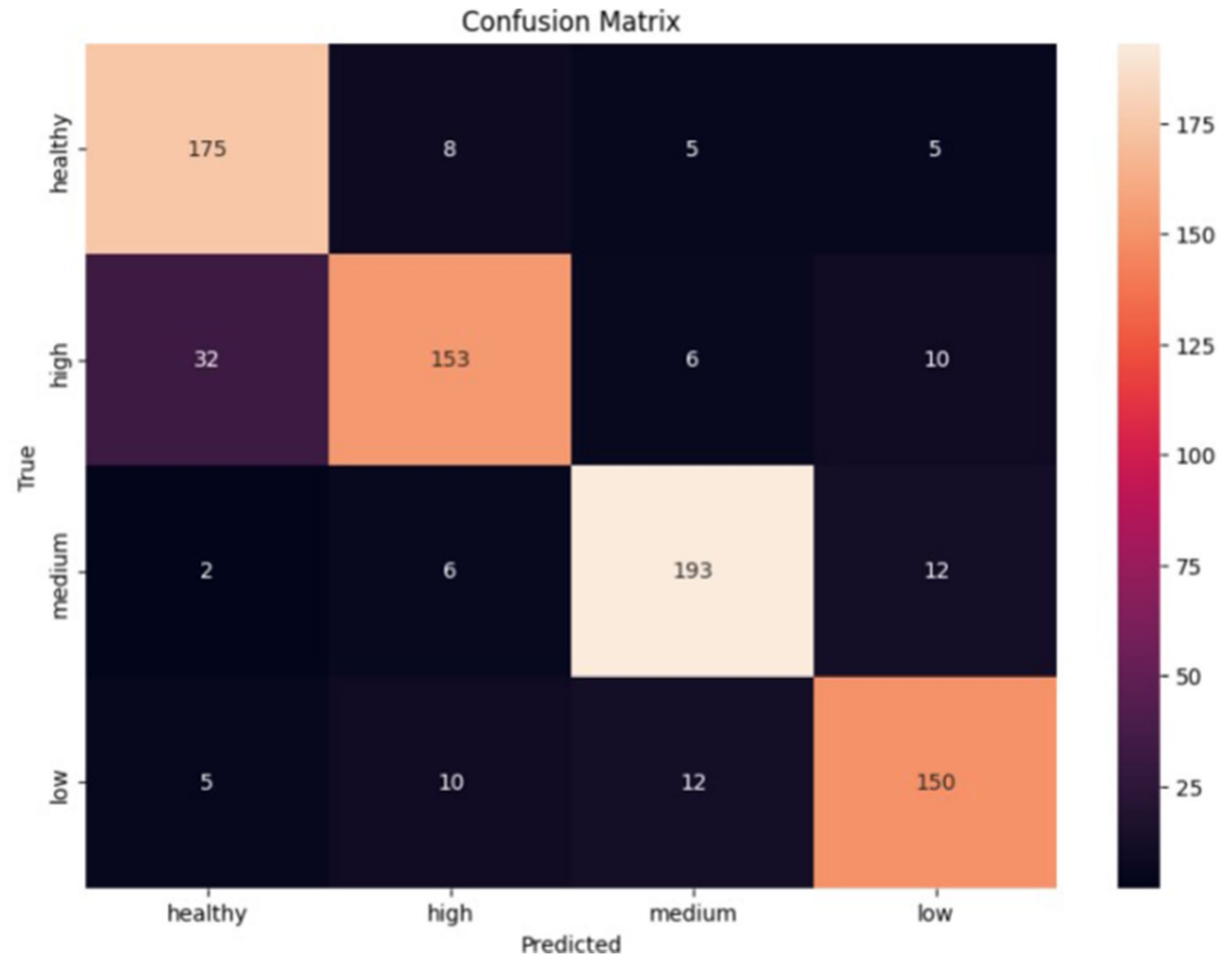
Error matrix of the LSTM classifier.

The accuracy of the training data starts at 46.11% in the first epoch and gradually increases with each epoch. The final accuracy is 90.94%. This shows that the model learns and improves its performance on the training data over time. The same is true for the validation data. In general, the model shows a positive trend in accuracy on both training and validation data, which indicates that the model is learning effectively and progressing in its task (see Figure 7).

The classifier made the most errors between the health and high classes, while the medium class was the best predicted. Given the errors, the classifier performed exceptionally well in classifying all classes.

## 5 Discussion

As a result of the experiments, both models coped with the task quite well, showing promising results in model classification. Based on the training results of the LSTM and CNN models, we can make the following observations:

– LSTM model accuracy:

On the training set: 0.9094.
On the validation set: 0.8652.
The LSTM model achieves reasonably high accuracy in the training and validation datasets. This indicates the model’s ability to learn and generalize dependencies in the data. However, it is worth noting that the accuracy on the validation set is slightly lower than on the training set, which may indicate a slight overtraining of the model.
– Accuracy of the CNN model:

On the training set: 0.9460
On the validation set: 0.8774.
The CNN model also achieves high accuracy on the training and validation datasets. The accuracy values on the training set are impressive, which may indicate that the model can learn well on the dataset. The accuracy of the validation set is also high, which shows the model’s ability to generalize the learned relationships to new data.

If compare the model data, it allows the following conclusions:

– The LSTM model shows higher accuracy on the training set but lower accuracy on the validation set than the CNN model.
– The CNN model has higher accuracy on the validation set, which may indicate a better ability to generalize to new data.
– Both models achieve high accuracy values, which indicates their effectiveness in audio classification.

For analysis, the results of using two models are represented in Table 2.

**Table 2:** Combined results of model testing.

Class	Metric	CNN	LSTM
Healthy	Precision	0.86	0.82
	Recall	0.95	0.91
	F1-score	0.90	0.86
Low	Precision	0.91	0.85
	Recall	0.95	0.85
	F1-score	0.93	0.85
Medium	Precision	0.93	0.89
	Recall	0.86	0.91
	F1-score	0.89	0.90
High	Precision	0.90	0.86
	Recall	0.82	0.76
	F1-score	0.86	0.81

Based on the test results in terms of metrics and error matrices, the following observations can be made:

– The CNN classifier has higher precision, recall, and F1-score values for the Healthy, Low, and Medium classes than the LSTM classifier.
– The LSTM classifier has higher precision, recall, and F1-score for the High class than the CNN classifier.
– Both classifiers generally demonstrate acceptable accuracy and efficiency in classifying audio data. However, there may be differences in performance for individual classes.
– The best results are achieved for the Healthy and Low classes in both models, with high precision, recall, and F1-score parameters.
– Both models have lower recall and F1-score for the High class, which may indicate problems in recognizing this class.
– The LSTM classifier has a higher precision for the Medium and High classes than the CNN classifier but a lower recall for the Medium class.
– The CNN classifier has a more stable accuracy for all classes than the LSTM classifier.
– The two classifiers made the most errors between the Health and High classes, which indicates that they are not sufficiently different for these representations

However, each model made the fewest errors differently: CNN made the fewest mistakes in determining the Low class, and LSTM made the fewest mistakes in classifying the Medium stage.

Lower precision in medium and high cases indicates that the model produces a higher number of false positives, potentially leading to unnecessary treatments or interventions. On the other hand, lower recall suggests a higher number of false negatives, meaning critical conditions could go undetected. A detailed error analysis should be conducted to determine the characteristics of misclassified samples. This includes:

- Identifying specific patterns or features that the model struggles with in medium and high cases.
- Analyzing whether these misclassifications are due to insufficient training data, feature representation, or model limitations.
- Evaluating cases where misclassifications occur more frequently (e.g., borderline cases between categories).

Future efforts will improve feature extraction and data preprocessing to address lower precision and recall in medium and high cases. Applying ensemble or hybrid models will provide the ability to make models more stable to a broader range of possible cases. We also see potential in extending the datasets, focusing on additional medium and high pathology samples, and augmenting them using synthetic data generation techniques. One of the essential parts of conducting future research and preparing production solutions is ethical considerations and applying fail-safe mechanisms, which will guide the minimization of missed diagnoses and ensure safe deployment.

## 6 Conclusion

This research aimed to address the critical issue of pronunciation disorders in post-traumatic military patients by applying artificial intelligence and machine learning. The study involved a comprehensive analysis of existing literature, selecting appropriate machine learning models, generating relevant training data, and implementing a specialized software architecture.

Two methods were used to transform the audio recordings: shallow spectrograms, which were used in conjunction with the CNN model, as this model works well with images, and shallow cepstral coefficients for the LSTM model, which works reasonably well with sequential numerical data.

The methods were evaluated and analyzed using loss and precision functions for training and validation data, error matrices, and precision, recall, and F1-score metrics. It can be concluded that both models coped quite well with the given task, showing overall accuracies of 94% (CNN) and 91% (LSTM).

Both classifiers generally demonstrate acceptable accuracy and efficiency in classifying audio data. However, there may be differences in performance for individual classes. The best results for both models’ Healthy and Low courses are achieved with high precision, recall, and F1-score parameters.

According to the results of the research and the indicators obtained from the selected metrics, it can be claimed that these methods are effective for the analysis and detection of this disease, which will perfectly classify the disease for further pronunciation correction.

The models proposed and used in this study were limited by the quality of the audio data and the computational resources available. This leads to its good effectiveness on the existing datasets, but it is not appropriate to apply to a wide range of datasets. In particular, noisy or incomplete recordings may slightly impact the performance of models. Moreover, the capability of both CNN and LSTM models to generalize different speech disorders or new data may need further validation. More testing with out-of-sample data will be essential to ensure robust performance across different patient groups.

The findings of this research underscore the potential of artificial intelligence and machine learning in addressing the rehabilitation needs of post-traumatic military patients with pronunciation disorders. Further research and refinement of the models could lead to enhanced practical applications and contribute significantly to the field of communication rehabilitation for individuals who have experienced trauma. The software module can be integrated into applications dealing with speech disorders.

## Data Availability

Publicly available datasets were analyzed in this study. This data can be found at: https://www.kaggle.com/datasets/iamhungundji/dysarthria-detection/data.

## Footnotes

^1^ https://www.kaggle.com/datasets/iamhungundji/dysarthria-detection/data
